# The Involvement of Renal Capsule Is Associated With Acute Kidney Injury in Patients With Acute Pancreatitis

**DOI:** 10.3389/fmed.2021.724184

**Published:** 2021-10-04

**Authors:** Mei Wei, Jingzhu Zhang, Cheng Qu, Yang Liu, Kun Gao, Jing Zhou, Lu Ke, Zhihui Tong, Weiqin Li, Jieshou Li

**Affiliations:** ^1^Center of Severe Acute Pancreatitis, Department of Critical Care Medicine, Jinling Hospital, Medical School of Nanjing University, Nanjing, China; ^2^Center of Severe Acute Pancreatitis, Department of Critical Care Medicine, Jinling Clinical Medical College of Nanjing Medical University, Nanjing, China; ^3^National Institute of Healthcare Data Science at Nanjing University, Nanjing, China

**Keywords:** acute pancreatitis, acute kidney injury, renal capsule, computed tomography, renal rim grade

## Abstract

**Background:** Acute pancreatitis (AP) is characterized by pancreatic/peripancreatic inflammation. Involvement of renal capsule refers to peripancreatic inflammation extending beyond the Gerota fascia and disappearance of renal rim sign (+) on CT images. However, its association with acute kidney injury (AKI), an important complication of AP, was rarely studied.

**Aim:** This study aimed to assess the relationship between the involvement of renal capsule and AKI in a cohort of patients with AP.

**Methods:** We retrospectively screened all the patients admitted for AP from January 2018 to December 2019. The involvement of renal capsule was judged by experienced radiologists according to the CT imaging. Propensity score matching (PSM) was used to control for biases in group sizes and baseline characteristics. The primary outcome was the development of AKI during the index admission. We also categorized the pararenal inflammation with the renal rim grade (RRG) and compared the incidence of AKI among different grades.

**Results:** Involvement of renal capsule was identified in 71 of 503 patients (14.1%). The incidence of AKI was significantly higher in these patients when compared with the matched controls (43/71, 60.6% vs. 12/71, 16.9%, *p* < 0.001). Moreover, mortality also differed between groups (12.7% vs. 1.4%, *p* = 0.017). Multivariable logistic regression showed that renal capsule involvement is an independent risk factor of AKI (odds ratio, 4.355; 95% confidence interval, 1.434, 13.230, *p* = 0.009). Patients with RRG grade III had a significantly higher incidence of AKI than the other two grades (60.6% for Grade III, 17.1% for Grade II, and 3.8% for Grade I, *p* < 0.001).

**Conclusion:** Involvement of renal capsule is associated with higher AKI incidence and mortality.

## Introduction

Acute pancreatitis (AP), an inflammatory disease of the pancreas, has varying clinical courses (1). Acute kidney injury (AKI) is common during AP and is associated with the severity of AP (1, 2). Devani et al. found that the mortality among patients with AP who developed AKI was significantly higher than those without (8.8% vs. 0.7%; *p* < 0.01) in a propensity-matched analysis (3).

Mechanisms for the development of AKI in AP are multifactorial, such as increased vascular permeability and hypovolemia, local inflammation, renal vasoconstriction, intravascular coagulation, and drug-related nephrotoxic effects (2). During AP, enzyme-rich collections of inflammatory fluid could involve retroperitoneal and peritoneal spaces extensively (4). Anatomically, the hilum of the right kidney lies posterior to the head of the pancreas, and the upper pole of the left kidney is posterior to the tail of the pancreas. Thus, pancreatic effusions could easily extend to pararenal and perirenal spaces, potentially leading to inflammation-related renal injury (1, 2, 5).

As previously described by Imamura et al. (6), the involvement of the kidney and surrounding tissues can be categorized by the renal rim grade (RRG) based on imaging features. Grade I is defined as no increase in CT attenuation of the anterior pararenal spaces and perirenal spaces, while Grade II refers to an increase in the CT attenuation of the pararenal space (pancreatic side of the Gerota fascia) but without change in the perirenal space. Grade III is defined when the peripancreatic inflammation extends beyond the Gerota fascia, namely, the renal capsule gets involved. Furthermore, Imamura et al. demonstrated that the RRG was useful to assess the severity of AP. However, the association between the involvement of renal capsules and the evolution of kidney dysfunction was not assessed.

In the present study, we aimed to assess the association between renal capsule involvement and the development of AKI in a cohort of patients with AP.

## Methods

### Study Design

This is a retrospective, single-center study. Patients admitted to the Center of Severe Acute Pancreatitis (CSAP), Jinling Hospital, from January 1, 2018 to December 31, 2019, were screened for eligibility. All the data were extracted from an electronic database (Acute Pancreatitis Database, AP Database), which prospectively collected clinical data of the study patients. Informed consent for data collection and academic use was obtained from the patients during hospitalization. This study was approved by the management committee of the AP Database (no: 2020 JLAPDMC-007).

### Participants

Acute pancreatitis was diagnosed according to the Revised Atlanta Classification (RAC) (7) when meeting at least two of the following three criteria: (1) characteristic abdominal pain of AP; (2) serum amylase and/or lipase >3 times the upper limit of normal; and (3) characteristic imaging findings of AP.

Inclusion criteria are as follows: (1) admission within 14 days from onset of abdominal pain and (2) age ≥ 18 years, and exclusion criteria are as follows: (1) infected pancreatic necrosis confirmed before admission; (2) CT data unavailable; (3) chronic pancreatitis; (4) pancreatic tumors; (5) recurrent AP within 1 year; (6) a history of chronic kidney disease (abnormalities of kidney structure or function for at least 3 months regardless of the underlying cause, such as markers of kidney damage (albuminuria, urinary sediment abnormalities, electrolyte and other abnormalities due to tubular disorders, abnormalities detected by histology or imaging, or history of kidney transplantation) or decreased glomerular filtration rate [GFR, less than 60 ml/min per 1.73 m^2^) (8)] before AP onset; and (7) pregnancy.

### CT Imaging and Definitions

All the imaging data (plain scan and enhanced scan) of the study patients were obtained using Siemens Somatom Definition dual-source CT. The parameters were as follows: tube voltage, 120 kV; tube current, 230 mA; section thickness, 1/1.5 mm; rotation time, 0.5 s, and pitch, 1.2. After a plain scan, contrast-enhanced imaging was performed with an iodinated contrast agent (ioversol) administered intravenously using a high-pressure syringe (1.5 ml/kg, 3–4 ml/s). The arterial and portal phases were conducted with a post-injection delay of 30 and 60 s, respectively. The delay scan was obtained at 180 s after injection of contrast agent if necessary. CT images were reviewed by two independent radiologists who were blinded to the clinical information of the patients. According to CT imaging findings, patients were categorized by the RRG (6). All disagreements were solved through discussion until consensus was reached. Patients with RRG III were deemed to have renal capsule involvement.

Acute kidney injury was defined by an abrupt decrease in kidney function based on the Kidney Disease Improving Global Outcomes (KDIGO) consensus criteria (9) and staged by increased serum creatinine and decreased urine output.

Major adverse kidney events within 30 days (MAKE30) are a composition of in-hospital mortality, receipt of new renal replacement therapy (RRT), or persistent renal dysfunction within 30 days of admission or before the index discharge, as reported by Semler et al. (10).

### Data Collection

All the demographic characteristics, laboratory data, and imaging data were extracted from the AP Database. Baseline characteristics included age, gender, body mass index (BMI), etiology, comorbidity, time from onset of symptoms to admission, and sequential organ failure assessment (SOFA) score at admission. Laboratory data included consecutive blood urea nitrogen (BUN) and blood creatinine levels after admission. The arterial phase was used for assessing the computed tomography severity index (CTSI) (11), which could describe the degree of pancreatic/peripancreatic damage, including inflammation and necrosis, and define the RRG. Moreover, we also collected clinical outcomes, including the development of AKI, mortality, other major complications (bleeding and infected pancreatic necrosis), and interventions.

### Study Outcomes

The primary outcome of the study is the incidence of AKI during the index admission. Readmission within 3 days after the index discharge will be counted as the same admission. All the secondary outcomes were also registered within the index admission, including mortality, length of intensive care unit (ICU) stay, organ failure, abdominal bleeding, infected pancreatic necrosis, and interventions. Data for MAKE30 were collected before the index discharge or 30 days after admission, whichever happened first.

### Statistical Analysis

Propensity score generation and matching were performed using the R package Match It (R Foundation for Statistical Computing, Vienna, Austria). Briefly, the propensity score was generated separately for patients with or without the involvement of renal capsules using a logistic regression model derived from four clinical variables (gender, age, etiology, and time from onset of symptoms to admission). Following propensity score generation, the two groups were matched using 1:1 nearest neighbor (Greedy-type) matching and a caliper width of 0.1 SD of the propensity score logit. Matching was performed without replacement, and non-matched results were discarded. Improvement in covariate balance following matching was measured using conditional logistic regression and conditioned on the specific pair identification assigned to each match. Continuous data were expressed as mean ± SD or median ± interquartile range (IQR), depending on its distribution. Categorical variables were described as frequency (percentage). As for analysis, paired-samples *t*-test and Wilcoxon rank-sum test were applied for continuous variables, while a chi-square test and Fisher's exact test were used in categorical variables. Apart from age, gender, and etiology of AP, variables were included in the multivariable logistic analysis if *p* < 0.1 in univariable logistic regression. The results were presented as odds ratio (OR) and 95% confidence interval (CI). All the analyses were completed by SPSS 22.0. Significance was defined as *p* < 0.05 (2-sided).

## Results

### Demographic and Clinical Characteristics of Patients

**Figure 1:**
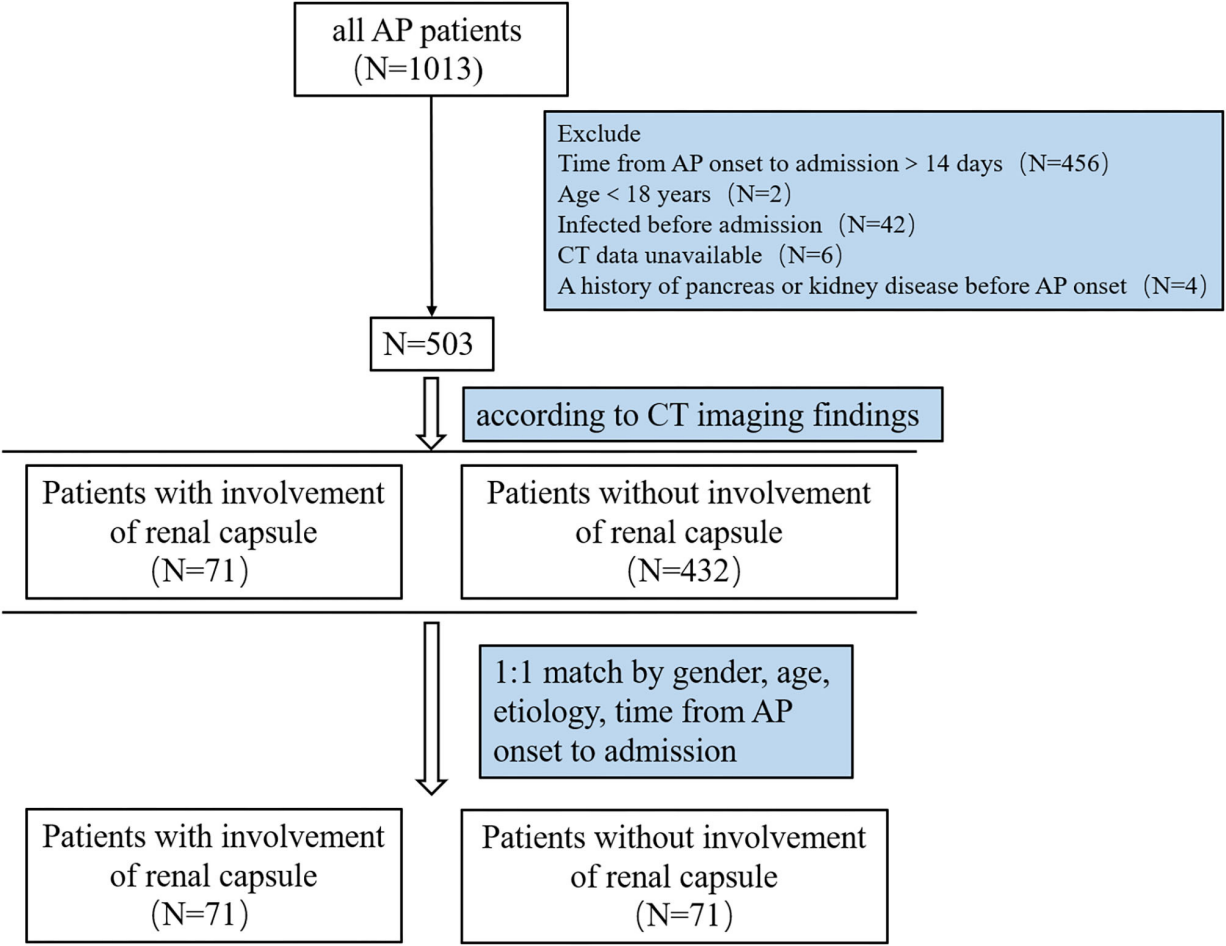
Flow chart of patients with AP in the study. AP, acute pancreatitis; CT, computed tomography.

**Figure 2:**
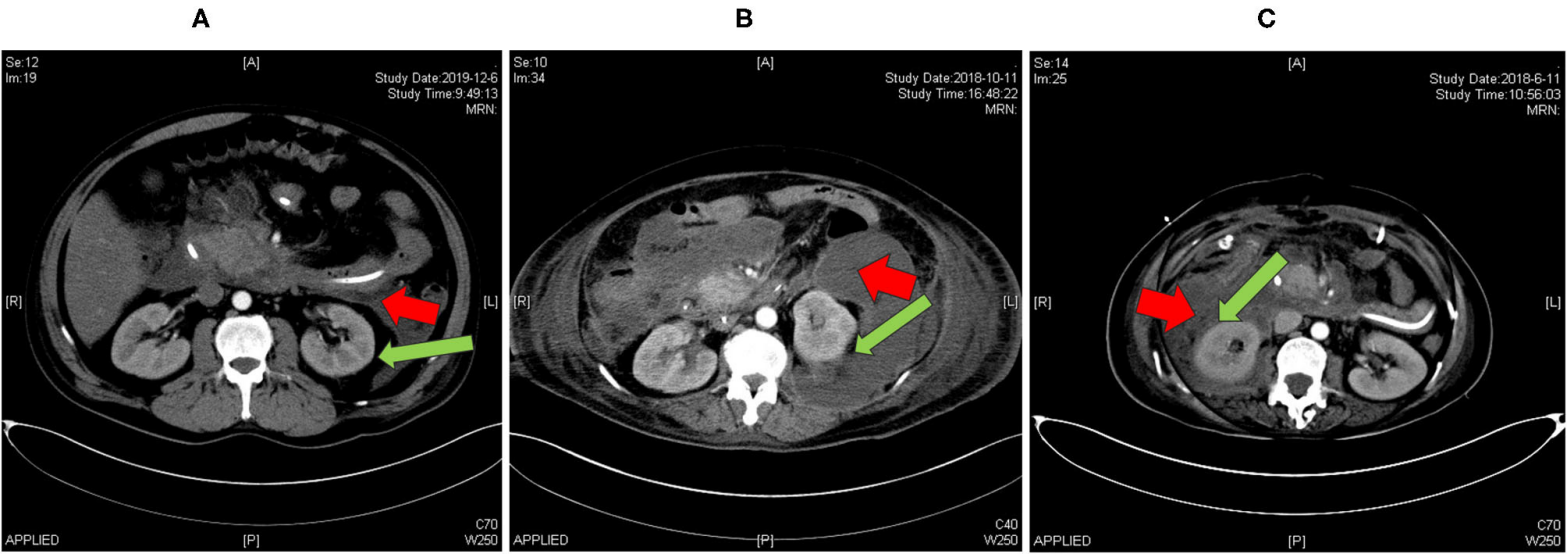
Abdominal CT imaging (arterial phase) of patients with AP included in our study. **(A)** A patient admitted to hospital in December 2019 with no involvement of renal capsule (RRG II); **(B)** a patient admitted to hospital in October 2018 with partial involvement of renal capsule (RRG III); **(C)** a patient admitted to hospital in June 2018 with full involvement of renal capsule (RRG III). Red arrow: pancreatic collections, green arrow: renal capsule. RRG, renal rim grade.

Among 1,013 patients screened, 503 were included for analysis (Figure 1). A total of 71 patients had renal capsule involvement (typical CT features are shown in Figure 2). Of these, 25.4% (18/71) presented with involvement of the left renal capsule alone, 45.1% (32/71) presented with involvement of the right capsule alone, and 29.6% (21/71) presented with involvement of both the left and right capsules (Table 1). After propensity score matching (PSM), we got 71 control patients in pairs with the study patients (Figure 1 and Supplementary Figure S1).

**Table 1:** Differences in demographic and baseline clinical characteristics between the two AP groups.

**Variable**	**Control group** **(*n* = 71)**	**Involvement of renal capsule** **(*n* = 71)**	***p*-value**
Age, yr, mean ± SD	42.70 ± 10.176	42.44 ± 9.711	*p* = 0.730
Gender (male), *n* (%)	43 (60.6%)	44 (62.0%)	*p* = 0.863
BMI, kg/m^2^, mean ± SD	26.00 ± 3.28	27.25 ± 5.12	*p* = 0.293
Etiology, *n* (%)			*p* = 0.714
Biliary	18 (25.4%)	19 (26.8)	
Hyperlipidemia	50 (70.4%)	50 (70.4%)	
Alcoholic	1 (1.5%)	2 (2.8%)	
Idiopathic	2 (2.8%)	0 (0.0%)	
Comorbidities, *n* (%)			
Hypertension	13 (18.3%)	15 (21.1%)	*p* = 0.673
Fatty liver	23 (32.4%)	16 (22.5%)	*p* = 0.188
Diabetes	17 (23.9%)	18 (25.4%)	*p* = 0.846
Time from onset of symptoms to admission, d, median (IQR)	4 (2, 7)	5 (3, 10)	*p* = 0.134
Time to CT imaging, d, median (IQR)	1 (1, 2)	1 (1, 2)	*p* = 0.932
SOFA, median (IQR)	3 (2, 5)	4 (2, 7)	*p* = 0.230
CTSI, median (IQR)	4 (3, 6)	6 (4, 8)	*p* < 0.001
Types of involvement			
Only left, *n* (%)	—	18 (25.4%)	
Only right, *n* (%)	—	32 (45.1%)	
Both sides, *n* (%)	—	21 (29.6%)	

*SOFA, sequential organ failure assessment; CTSI, computed tomography severity index*.

Demographic and baseline clinical characteristics are presented in Table 1. There were no significant differences in age (42.70 ± 10.176 vs. 42.44 ± 9.711 years, *p* = 0.730), gender (male%, 60.6% vs. 62.0%, *p* = 0.863), BMI (26.00 ± 3.28 vs. 27.25 ± 5.12 kg/m^2^, *p* = 0.293), and time from onset of symptoms to admission (median 4 (IQR: 2, 7) vs. 5 days (IQR: 3, 10), *p* = 0.134) between the two groups.

The median levels of BUN (7.1 vs. 4.0 mmol/L, *p* < 0.001) and creatinine (86.0 vs. 52.8 μmol/L, *p* < 0.001) at admission were significantly higher in the patients with renal capsule involvement. Moreover, they had a higher CTSI score [6 (IQR: 4, 8) vs. 4 (IQR: 3, 6), *p* < 0.001]. However, the two groups were comparable in terms of the SOFA score at admission (Table 1).

### Primary and Secondary Outcomes

**Figure 3:**
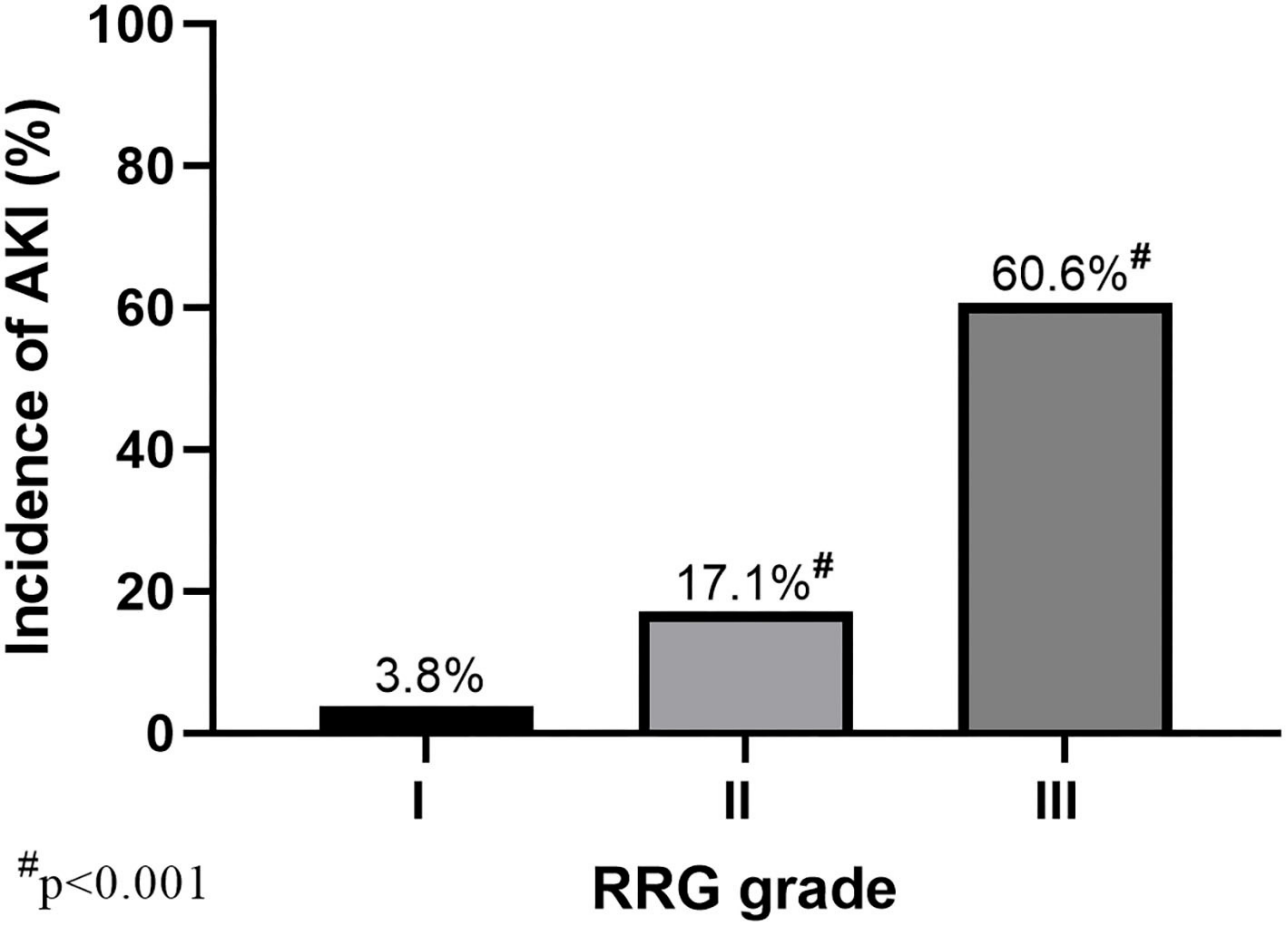
AKI incidence with different RRG grades of the kidney (RRG I–III). A total of 503 patients with AP were divided into three grades based on CT images: RRG I (*N* = 52), RRG II (*N* = 380), and RRG III (*N* = 71). AKI, acute kidney injury; AP, Acute pancreatitis; RRG, renal rim grade.

The primary and secondary outcomes are shown in Table 2. The incidence of AKI in patients with renal capsule involvement (RRG III) was 60.6% (43/71), which was significantly higher than those without renal capsule involvement (12/71, 16.9%; Table 2 and Figure 3). For MAKE30, patients with renal capsule involvement had significantly more adverse events than the controls (57.7% vs. 16.9%, *p* < 0.001) within 30 days, including in-hospital mortality (9.9% vs. 1.4%, *p* = 0.069), receipt of new RRT (57.7% vs. 16.9%, *p* < 0.001), and persistent renal dysfunction (8.5% vs. 0, *p* = 0.037).

**Table 2:** Differences in primary and secondary outcomes between the two AP groups.

**End point**	**Control group** **(*n* = 71)**	**Involvement of renal capsule** **(*n* = 71)**	***p*-value**
Primary outcome			
AKI occurrence, *n* (%)	12 (16.9%)	43 (60.6%)	*p* < 0.001
Secondary outcome			
MAKE30, n (%)	12 (16.9%)	41 (57.7%)	*p* < 0.001
Persistent kidney dysfunction plus death, *n* (%)	1 (1.4%)	15 (21.2%)	*p* < 0.001
ICU mortality, *n* (%)	1 (1.4%)	9 (12.7%)	*p* = 0.017
ICU duration, d, median (IQR)	5 (3, 11)	14 (7, 30)	*p* < 0.001
Organ failure			
ARDS, *n* (%)	15 (21.1%)	45 (63.4%)	*p* < 0.001
Sepsis, *n* (%)	2 (2.8%)	15 (21.1%)	*p* = 0.001
Septic shock, *n* (%)	2 (2.8%)	13 (18.3%)	*p* = 0.003
Other complications			
IPN, *n* (%)	3 (4.2%)	16 (22.5%)	*p* = 0.001
Abdominal bleeding, *n* (%)	0 (0)	9 (12.7%)	*p* = 0.006
Interventions			
PCD, *n* (%)	3 (4.2%)	12 (16.9%)	*p* = 0.014
Endoscopic necrosectomy, *n* (%)	0 (0)	3 (4.2%)	*p* = 0.243
Surgery, *n* (%)	1 (1.4%)	6 (8.5%)	*p* = 0.121

*AKI, acute kidney injury; MAKE30, major adverse kidney events within 30 days; ICU, intensive care unit; ARDS, acute respiratory distress syndrome; IPN, infected pancreatic necrosis; PCD, percutaneous catheter drainage*.

During the index admission, mortality was significantly higher in patients with renal capsule involvement (12.7% vs. 1.4%, *p* = 0.017). Among the deceased patients, nine patients died of septic shock-related multiple organ failure, and one died of hepatic or septic encephalopathy. Besides, the ICU duration was significantly longer in patients with involvement of renal capsule when compared to the control group [14 (IQR: 7, 30) vs. 5 days (IQR: 3, 11), *p* < 0.001]. Moreover, patients with renal capsule involvement had a higher incidence of multiple complications, such as acute respiratory distress syndrome (ARDS), sepsis, septic shock, and infected pancreatic necrosis.

Risk factors for AKI in univariate and multivariate logistic regression are shown in Table 3. Renal capsule involvement was associated with AKI in univariate logistic regression (OR, 7.551; 95% CI, 3.454, 16.507; *p* < 0.001). After adjusting for age, gender, etiology, CTSI, BUN, and neutrophils, renal capsule involvement was found to be an independent risk factor for AKI (OR, 4.355; 95% CI, 1.434, 13.230; *p* = 0.009).

**Table 3:** Risk factor analysis for AKI in univariate and multivariable logistic regression.

	**Univariate**			**Multivariate**		
	**OR**	**95% CI**	***p*-value**	**OR**	**95% CI**	***p*-value**
Age, yr	1.024	0.989, 1.060	0.185	1.010	0.961, 1.062	0.696
Gender (male)	1.337	0.664, 2.695	0.416	0.728	0.232, 2.292	0.588
Etiology			0.454			0.040
Biliary	0.320	0.047, 2.176	0.244	0.037	0.003, 0.516	0.014
Hyperlipidemia	0.444	0.071, 2.780	0.386	0.144	0.014, 1.429	0.098
Renal capsule involvement	7.551	3.454, 16.507	<0.001	4.355	1.434, 13.230	0.009
CTSI	1.423	1.195, 1.694	<0.001	1.270	0.971, 1.662	0.081
BUN, mmol/L	1.542	1.316, 1.808	<0.001	1.462	1.226, 1.742	<0.001
Neutrophils (%)	1.052	0.993, 1.115	0.086	1.041	0.950, 1.141	0.385

*AKI, acute kidney injury; OR, odds ratio; CI, confidence interval, CTSI, computed tomography severity index; BUN, blood urea nitrogen*.

The results of an additional subgroup analysis (patients with renal capsule involvement) are shown in Supplementary Table S1. Among them, patients who developed AKI had a higher SOFA score [6 (3, 9) vs. 2 (2, 3.75), *p* < 0.001] at baseline when compared with those who did not. Apart from that, some other common laboratory markers, such as lymphocytes, procalcitonin (PCT), and blood glucose, also differed between groups (*p* = 0.025, *p* < 0.001, and *p* = 0.003, respectively).

## Discussion

In this study, we found that renal capsule involvement was common and associated with the development of AKI and poor clinical outcomes in patients with AP. The association remained tenable after adjustment for demographic and disease characteristics.

The integrity of the renal structure and its surrounding tissues is essential for multiple functions, such as forming an effective filtration barrier, maintaining fluid and ion homeostasis (12–14). A variety of diseases are marked by the involvement of the renal structure and its surrounding tissues (6, 15–17). McCort et al. reported a sign of the infiltration of perirenal fat by hemorrhage on abdominal CT imaging during renal parenchyma laceration (16). Fritzsche et al. (17) reported six cases, such as inflammatory diseases or different tumors, in which perirenal fat was infiltrated by inflammatory fluid and tumor when violated. Anatomically, the renal capsule refers to a thin membranous sheath covering the external surface of the kidney (18), surrounded by perirenal adipose tissue. It has been demonstrated that pancreatic inflammation could cause lipolysis when it leaks into visceral adipose tissue in AP mice, worsening organ failure (19). Thus, the inflammatory involvement of the renal capsule always involved the adipose tissue around the capsule, thereby resulting in lipolysis. In view of this, our results support the idea that involvement of renal capsule in AP could lead to or aggravate renal dysfunction, and the mechanism might be complex (2, 20). Inflammatory effusions could damage renal or vascular epithelial cells, thereby leading to an increase in oxidative stress, mitochondrial injury, and production of reactive oxygen species (ROS) as a result of exposure to damage-associated and pathogen-associated molecular patterns it contained (21). Inflammation-triggered lipolysis of the adipose tissue could further cause renal injury by leading to the elevation of acute non-esterified fatty acid (NEFA), which could inhibit mitochondrial function (22, 23).

Involvement of renal capsule in AP was reported first by Dembner (24), and since then, there have been several reports regarding its clinical implications (4, 5, 25). Imamura et al. (6) established the RRG score to categorize this phenomenon based on CT imaging and deemed renal capsule involvement as the most severe grade. In addition, Li et al. (26) found that extrapancreatic inflammation on CT score (EPIC) correlated with poor prognosis in AP, which is consistent with Wang et al.'s (15, 27) findings in more severe cases of AP. Different from the previous studies that took the three-category RRG score or more extensive EPIC score as severity predictors, we mainly focus on the clinical implication of renal capsule involvement, namely, RRG III. Accordingly, we design the study methodology, dividing patients based on the presence of renal capsule involvement. To compensate for demographic and clinical imbalances between groups, we adopted PSM, making the two groups more comparable. Our results indicated that renal capsule involvement is of great clinical significance, suggesting approximately four times higher rates of AKI and a higher risk of death. Moreover, to make the results more comparable with studies conducted in other populations like critically ill patients (28), we added the MAKE30 (10) as a secondary endpoint. We found that the renal capsule involvement was also associated with renal recovery (more persistent renal dysfunction), which may guide future studies focusing on the recovery of AKI.

Computed tomographic imaging is an important tool for diagnosing and managing AP, which is convenient and able to provide insight into pancreatic inflammation and necrosis (29). Since renal capsule involvement is easy to recognize on CT imaging, it is of potential use in identifying AP patients at high risk of developing AKI and facilitating timely treatment. Moreover, within patients with renal capsule involvement, there were significant differences (30) in laboratory biomarkers between patients who developed AKI and those who did not, such as lymphocytes, PCT, and glucose. It indicates that CT imaging of renal capsule involvement in combination with clinical markers may help identify patients with AKI with better accuracy. However, due to the observational nature of this study, we could not test that in the present study. A future prospective study is worth testing this possibility.

There are several limitations to our study. First of all, due to its retrospective design, the only association could be demonstrated from our results. Moreover, as AKI could occur very early during the disease course, it is hard to determine whether the involvement of renal capsule contributed to AKI, which is why we include a more objective and easy-to-measure outcome measure, MAKE30. The strengths of this study include its matched design and accurate assessment by radiologists blinded to the severity of AP.

In conclusion, the involvement of renal capsules is common in AP and associated with the development of AKI. It may help identify patients at high risk of AKI, thereby facilitating appropriate treatment. Prospective studies are needed to assess the causal relationship between renal capsule involvement and kidney dysfunction.

## Data Availability Statement

The raw data supporting the conclusions of this article will be made available by the authors, without undue reservation.

## Ethics Statement

The studies involving human participants were reviewed and approved by the management committee of the AP Database (No: 2020 JLAPDMC-007). The patients/participants provided their written informed consent to participate in this study. Written informed consent was obtained from the individual(s) for the publication of any potentially identifiable images or data included in this article.

## Author Contributions

MW and JZ contributed to design, data collection, data analysis, and manuscript drafting. CQ and YL contributed to data collection and analysis. KG and JZ contributed to data analysis and presentation. LK and ZT contributed to design and manuscript drafting. WL and JL contributed to the study design and revision. All the authors have read and approved the final manuscript.

## Funding

This study was supported by the National Natural Science Foundation of China (no. 81900592) and Key Research and Development Program Foundation of Jiangsu Province of China (no. BE2016749).

## Conflict of Interest

The authors declare that the research was conducted in the absence of any commercial or financial relationships that could be construed as a potential conflict of interest.

## Publisher's Note

All claims expressed in this article are solely those of the authors and do not necessarily represent those of their affiliated organizations, or those of the publisher, the editors and the reviewers. Any product that may be evaluated in this article, or claim that may be made by its manufacturer, is not guaranteed or endorsed by the publisher.
