# Supplementary material for: The Involvement of Renal Capsule Is Associated With Acute Kidney Injury in Patients With Acute Pancreatitis
Source: Front Med (Lausanne). 2021 Oct 4;8:724184. doi: 10.3389/fmed.2021.724184 (PMC8520945; doi:10.3389/fmed.2021.724184)
Supplement: Supplementary file 1 [file Data_Sheet_1.pdf]

Table S1 Characteristic of patients with renal capsule involvement

	Non-AKI (N=28)	AKI (N=43)	p value
Gender (male), n (%)	16 (57.1%)	28 (65.1%)	0.499
Age, yr, mean±SD	41.1±8.925	43.3±10.199	0.356
BMI, kg/m2, median (IQR)	24.3 (20.7, 27.5)	27.4 (24.3, 31.1)	0.019
WBC, 10^9/L, median (IQR)	9.7 (6.3, 17.1)	10.6 (7.8, 16.9)	0.338
Lymphocytes, %, median (IQR)	9.6 (6.8, 11.8)	6.8 (4.9, 10.0)	0.025
Neutrophils, %, median (IQR)	84.6 (80.4, 88.2)	86.2 (81.3, 90.5)	0.206
Albumin, g/L, median (IQR)	27.8 (25.1, 30.5)	31.1 (26.9, 33.1)	0.033
Cholinesterase, kU/L, median (IQR)	3.9 (3.3, 5.3)	3.7 (3.0, 4.9)	0.371
Triglycerides, mmol/L, median (IQR)	3.3 (2.3, 7.1)	4.4 (2.7, 5.9)	0.491
Glucose, mmol/L, median (IQR)	7.8 (5.5, 10.8)	10.5 (7.9. 14.9)	0.003
PCT, ug/L, median (IQR)	0.7 (0.5, 1.8)	4.8 (2.0, 16.7)	<0.001
BUN, mmol/L, median (IQR)	4.3 (3.0, 6.1)	10.7 (6.9, 18.5)	<0.001
Creatinine, umol/L, median (IQR)	51.8 (41.7, 60.8)	224.6 (91.3, 337.0)	<0.001
BUN/creatinine, %, median (IQR)	0.08 (0.06, 0.11)	0.05 (0.04, 0.07)	<0.001
CRP, mg/L, median (IQR)	184.6 (143.2, 219.2)	174.3 (127.5, 239.3)	0.711
SOFA, median (IQR)	2 (2, 3.75)	6 (3, 9)	<0.001
CTSI, median (IQR)	6 (4, 6)	6 (4, 10)	0.412

Abbreviation: AKI, acute kidney injury; BMI, body mass index; WBC, white blood cell; PCT, procalcitonin; BUN, blood urea nitrogen; CRP, C-reactive protein; SOFA, sequential organ failure assessment; CTSI, computer tomography severity index

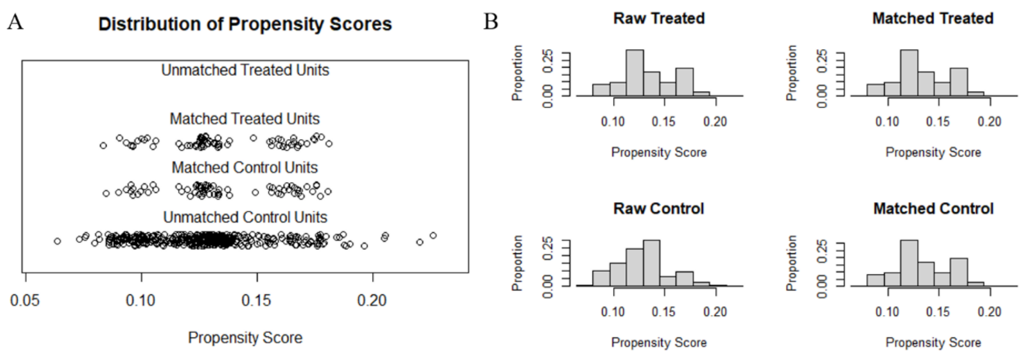

Figure S1. Propensity score matching jitter plot and the propensity score matching histogram. A. propensity score matching jitter plot; B. propensity score matching histogram
